# Taking a Gamble for High Rewards? Management Perspectives on the Value of Mental Health Peer Workers

**DOI:** 10.3390/ijerph15040746

**Published:** 2018-04-13

**Authors:** Louise Byrne, Helena Roennfeldt, Peri O’Shea, Fiona Macdonald

**Affiliations:** 1 Midwifery and Social Sciences, School of Nursing, Central Queensland University, 554/700 Yaamba Road, Norman Gardens, QLD 4701, Australia; h.roennfeldt@griffith.edu.au (H.R.); peri.oshea@yahoo.com.au (P.O.); 2 School of Management, RMIT University, 124 La Trobe St, Melbourne, VIC 3000, Australia; fiona.macdonald@rmit.edu.au

**Keywords:** service users, peer work, lived experience work, management, diversity employment, recovery, mental health services, mental health reform

## Abstract

Mental health peer work is attracting growing interest and provides a potentially impactful method of service user involvement in mental health design and delivery, contributing to mental health reform. The need to effectively support this emerging workforce is consequently increasing. This study aimed to better understand the views of management in relation to peer work and specifically explores the value of peer work from the perspective of management. This qualitative research employed grounded theory methods. There were 29 participants in total, employed in both peer designated and non-peer designated management roles, in not for profit and public health organisations in Queensland, Australia. The value of peer work as described by participants is found to be partially dependent on practical supports and strategies from the organisation. There were high benefits for all facets of the organisation when effective recruitment and ongoing support for peer workers was prioritised and a higher perception of limitations when they were not. Due to some parallels, it may be useful to explore the potential for peer work to be conceptually and/or practically considered as a form of diversity and inclusion employment.

## 1. Introduction

In the mental health sector, service user participation has gained impetus in recent decades as a critical component of mental health reform. Service user participation in the design and delivery of mental health services is now enshrined in public policy in the Western World, and is a significant feature of the contemporary ‘recovery’ approach in mental health. The recovery approach developed as a result of self-advocacy on the part of service users and was later also championed by allies from within the sector, presently the dominant new direction in mental health service delivery [1]. The central recovery concept is that a person experiencing significant mental health challenges can create a life of meaning that is chosen and directed by them, regardless of diagnosis or symptoms [2]. Recovery focuses on a holistic approach emphasising hope, autonomy, informed choice, connection and the existing strengths of the service user [3]. Supported by these key directives, service user participation manifests in a variety of ways within diverse settings. While informal, voluntary feedback on services still occurs, there is an increasing trend towards formal, paid positions for service users. In these positions—often referred to as ‘peer’ roles—people with a lived experience of mental health diagnosis, service use and periods of healing are employed to work from the perspective of their experiences, providing unique understanding and skills [4]. Peer roles encompass representative roles requiring periodic input, as well as part-time, casual and full-time positions, including direct support, systemic advocacy, management, research, education, and training [5].

Research findings suggest the use of peers can reduce overall service costs, lead to better recovery orientation and support a greater focus on person-directed care [6,7]. Peer workers reportedly possess a stronger natural focus on recovery and do not experience the same problems with competing clinical and professional priorities and perspectives that challenge traditional workers [8]. Research with service users has found peer workers can inspire hope and enable more equitable relationships between services and users [9,10]. While some traditional workers are reported to discriminate against both peer workers [11] and service users [12], the presence of peer workers can lead to increased understanding and empathy with people with a mental health diagnosis among colleagues in traditional roles [13,14,15]. As service user participation in the form of peer roles has become better established, attention is being directed to what constitutes effective and meaningful employment and integration of peers.

In this context, the present study takes a qualitative approach to exploring the effectiveness of peer roles through the perspectives of people employed in management positions within mental health services in the state of Queensland, Australia. While early research indicates management may play an important role in ensuring effectiveness [16], to date there has been little research exploring management perspectives of peer roles.

This paper explores the perceived value and limitations of peer roles from the perspective of people employed in management roles and, drawing on participant experiences, identifies strategies to support and maximise the benefits of employing peers.

## 2. Materials and Methods

### 2.1. Research Design

A grounded theory approach was utilised in this study as it is well suited to areas where little is known about an issue [17] as with management perspectives of peer workers. The grounded theory emphasis on ‘grounding’ the findings in the data promotes accurate representation of participant’s views [18] which is especially important when the subject group is under-represented within research.

### 2.2. Ethics

This study was conducted in accordance with the Declaration of Helsinki [19]. Ethical approval for participants from the not for profit sector was gained from the Central Queensland University Human Research Ethics Committee on the 18 December 2015—H15/11-262. Approval for participants from the public sector was gained from The Prince Charles Hospital Human Research Ethics Committee on the 20 September 2016—HREC/16/QPCH/298. Each Health and Hospital Service within the public sector then required site specific approvals which were obtained from seven separate Ethical Research and Governance Committees. Details of Health and Hospital Services are not reported to protect participant confidentiality. Invitations to participate included information about the right to withdraw without penalty at any stage of the process, and all participants gave informed, written consent prior to participation. Following transcription of interviews, identifying information including names, place names, and other unique data was removed or replaced with codes including pseudonyms. Coded transcripts were stored separately to identified material in locked, password protected files. 

### 2.3. Participation and Recruitment

As per ethics approvals, before invitations to participate were sent, CEO/Executive Directors of potentially relevant services/programs were asked to provide written permission for staff to be invited to participate. Approximately 100 not for profit and public health services were initially contacted by telephone from a database created by the research team, of all potentially relevant mental health services in the state of Queensland. During the initial contact, organisations were able to self-identify if they wished to receive a CEO letter. Of those choosing not to be contacted, reasons given were, already participating or recently participated in a research project, and/or excessive workload/limited staffing and a desire not to over-burden staff. From the 100 services initially contacted, a number were managed by the same umbrella organisation—resulting in some CEO letters allowing participation from multiple services. Other organisations were not deemed eligible as they did not provide mental health specific programs and had few or no identified mental health service users accessing the service.

All organisations that provided mental health specific services and/or assisted mental health service users that did choose to be contacted were sent a CEO letter. Forty-nine (49) CEO letters were sent and 24 CEO approvals were received. Invitations were sent to potential participants at all 24 organisations where approval was gained. In line with grounded theory, initial participants were purposively sampled, focusing on people employed within executive and senior management roles within mental health services. For the purposes of this study the definition of executive/senior management included: executive level staff; people with responsibility for allocation of staffing budgets and/or the ability to hire and fire; people with line management/supervisory responsibilities for peer staff. A total of 29 people from 24 organisations participated in the study; 16 participants employed within the not for profit sector and 13 employed in public health organisations. Some very large organisations, such as district health services and not for profit organisations with diverse services across the state included participation from more than one employee from different services within the organisation.

To gain diversity of perspectives, participants were invited from organisations with differing levels of peer employment, including no peer workers through to entirely peer-run services. In total, 20% of organisations had no history of employing peer workers, 44% of organisations had small numbers of peer staff and 36% had significant peer employment and experience with peer employment. Peers were employed in a variety of roles within organisations including systemic advocacy positions, education and training, and peer support workers.

Participants included people employed in traditional management roles (non-peer designated) including doctors, nurses, psychiatrists, psychologists, social workers, occupational therapists, executive administrators, and other health professionals. Other participants were employed in peer or carer designated management roles in both the public and not for profit sectors. Table 1. outlines the numbers of people employed in traditional roles and peer designated roles in both not for profit and government organisations.

### 2.4. Procedure

Twenty-five in-depth face-to-face interviews and one focus group were conducted. Six people attended the focus group and one focus group participant also chose to be interviewed. Each of the in-depth interviews was conducted either over the phone or in person by a single researcher. Interviews and the focus group were semi-structured with questions/topic areas covering participant views on the challenges and supportive factors for peer employment, differences in peer and traditional mental health work and the perceived value of peer roles. With participants’ consent, interviews were audio-taped.

### 2.5. Data Collection and Analysis

Participants for initial interviews were selected to represent the broadest range of relevant perspectives, in line with the open coding approach of grounded theory [20]. Participants from organisations with and without peer workers offered views on both the benefits and limitations of the peer roles. Employing an iterative cycle of data collection and analysis [21], once a small number of interviews had been conducted, they were analysed and the data emerging from those interviews was used to theoretically select the next participants. Initially all transcripts were coded using line-by-line analysis to identify emerging concepts. NVivo 11 coding software was utilised, but was not used to generate ‘computer coding’, rather as a means of organising and quickly accessing the study information, including coded transcripts and memos for identifying and separating researcher bias. The research team composed of three researchers, individually coded then swapped transcripts, providing feedback and input to the other transcripts and concept labels. At later stages of data collection and analysis, concepts were considered in relation to other emerging concepts and consequently clustered in categories according to their properties [21]. After data collection was complete, the research team discussed concepts, categories, and properties until consensus was reached. Results were then verified and confirmed with participants before being finalised.

To ensure different participant perspectives were represented, four participants were approached and provided verification from the following demographics; traditional management role within a not for profit organisation; peer designated management role within a not for profit; traditional management role within a public health organisation; peer designated management role within a public health organisation. Participant feedback was progressively incorporated, with the amended results presented to the next participant. From the first participant to provide feedback, only minor changes were requested, with all participants confirming the results accurately represented their experiences. The first participant requested the most minor amendments with requests decreasing, until the final participant requested no changes.

## 3. Results

Participants frequently raised the value of peer roles and benefits to services users and the organisation, as well as perceived limitations of the roles. They identified a range of strategies for maximising the benefits of the roles. For the purposes of reporting, participants have been assigned non-gender specific pseudonyms to assist in maintaining confidentiality.

### 3.1. Benefits to Service Users

All participants identified benefits of peer roles for service users, but those who employed peers were particularly enthusiastic. The unique skills and perspective of peer workers were considered to provide some benefits to service users that would not normally be the outcome of interaction with traditional support roles. Relationships between service users and peers were commonly perceived to be more equitable than relationships between service users and people employed in traditional roles. Peer workers’ ‘lived’ understanding was seen to provide unique credibility to the roles. Similarly, the inclusion of ‘lived experience’, ‘peer’, or ‘consumer’ within the job title was reported as providing positive opportunities for building rapport. Participants further described peer workers providing a living example of recovery and hope:

…we actually have people [peers] in our team [that] do hear voices, that still require hospitalisations at times …and they work and they have meaningful lives and they’re having those conversations with our consumers about that openly and people look at that and they go ‘wow’…what it does for people—‘ok this may not be my life forever, there’s actually hope here that something can change, even when things are really challenging’

—Larry

Other participants spoke of the ability of peers to empathise deeply or understand the experiences of service users, as well as having the capacity to initiate difficult or challenging conversations that may not have been effective coming from people employed in traditional roles. Peer workers were also seen to have a unique capacity to navigate the mental health system and provide practical strategies and knowledge that was of benefit to service users. Overall, the benefits to service users were considered evident in observed reductions in relapse, case management and rehospitalisation as a result of peer involvement, as in this example:

…[going from] people [service users] spending at least 70% of their time in hospital to having 0% hospital admission and at least 30% of them not even being case managed and we’re talking about people [who were] being case managed for 15 years and heavily case managed

—Bella

### 3.2. Benefits for the Organisation and Colleagues in Traditional Roles

In addition to unique benefits to service users, many participants outlined benefits to their team, service, or wider organisation as a result of peer employment. Some participants had utilised peer workers in a formal capacity to contribute to training for other staff. In less formal ways, peer workers were also considered to contribute to positive workplace culture:

…there is evidence that shows having a recovery support [peer] worker has driven a positive change, not just for the consumers [service users]—but within the clinical workforce. The culture of the clinical workforce in that team has changed

—Dorrell

Participants described the underpinning recovery framework as an important foundation for the culture of the organisation. Peer workers were seen to promote recovery and contribute to a stronger organisational commitment to recovery orientation. Participants reflected on how having people with an acknowledged lived experience in the team inspired more thoughtful use of language, ultimately leading to a more inclusive workplace and service. Participants further identified that peer workers were viewed as the barometer of recovery and helped hold an organisation accountable for their practice and language:

…this is the function of peer support, so you know it is this person’s job to make sure we’ve got consistent [recovery focused] language and that if they [peers] hear anything and it doesn’t sound like the correct sort of language we should be using, it’s their job to talk to the team about it

—Oscar

In addition to promoting recovery orientation, participants also described the specialised knowledge of peer workers and their role as a ‘bridge’ of understanding between workers in traditional support roles and service users. Workers in more traditional roles reportedly gained a better understanding of supporting people in crisis by seeking guidance from the lived understanding of peers. Peer roles were also seen to challenge prejudicial attitudes both within the community and organisations:

…it’s a deeper understanding of what the stigma issues are. Stigma’s not something that you just see, it’s something that’s ingrained, it’s something that’s in everybody’s psyche …I think it’s well worth going through the process [employing peer staff] even if it was only just to break down the stigma

—Josh

### 3.3. Limitations of Peer Work

Sometimes, the perceived value of the role was diminished by limitations. The emergent nature of peer work and, particularly, a lack of understanding from colleagues, purportedly contributed to the perception peer workers may pose a threat to existing roles:

…when we first started there was a lot of fear from the staff that ‘oh shit are they gonna be taking over our office’ you know ‘they better not be doing what we do’

—Sam

Despite significant increase in the development of peer positions, participants described the value of roles as variable across organisations, with some participants of the opinion poorly designed and supported peer positions could be tokenistic, consequently impacting their effectiveness.

…some of the positions themselves are, tokenistic is probably not an unfair word, or contrived in a way. There are also other organisations or other individuals for whom that [peer employment] really still is very much at the ‘you tick box’ kind of point

—Matt

Some participants spoke of limitations as arising from previous negative experiences with peer workers, particularly around the need for effective and responsible use of personal story. Participants described both positive experiences; where the peer workers had a good understanding of how to effectively use parts of their story to inspire hope, and less effective examples; where the peer worker was seen to focus too much on their story without using their experiences purposefully. Some participants also remarked on the fall-out when peers were not managing their own wellbeing adequately, subsequently impacting service users:

…if we have people [peer workers] talking to people [service users] about strategies for wellness absolutely they need to be able to manage their own. I’ve seen it have a very detrimental impact when we have an employee who was unwell talking about their stress and their un-wellness and their frustrations in front of consumers [service users]…they’re not coming here to listen to somebody else’s problems

—Larry

Many participants raised the idea that people in peer roles may be unreliable or become unwell as a barrier to engaging peers. This was an issue of dissension between participants, with some seeing the potential of peers becoming unwell as a risk, and others viewing it as a negative assumption or ‘myth’ not evidenced by their own experiences. Of note, participants with significant experience employing peers typically did not consider staff absenteeism an especial issue for peer workers. On the contrary, some participants described peer workers as demonstrating greater capacity for self-management than traditional workers:

...the peers are actually more proactive which I think is great because they know themselves and they know their limitations whereas sometimes some other staff [in traditional roles] think they’re ok and they’re not as attuned or aware of their own limitations

—Sarah

Similarly, rather than viewing peers as especially susceptible to burnout, the life experiences of peer workers were seen by some to increase resilience to workplace stress. The idea was also raised that peer roles being ‘out’ about their mental health challenges was preferable to the many people employed in the sector who did not disclose. Some participants compared the needs of staff with mental health diagnoses to those with physical health conditions, seeing no difference between accommodating occasional breaks from work for either.

Other participants described varied success employing peers, especially in relation to retention and absenteeism. Retention was often described as an issue at earlier stages of peer employment.

### 3.4. Practical Strategies and Supports

Many participants discussed the importance of practical strategies and supportive measures to maximise the effectiveness and consequently the benefits of peer roles, aiding sustainability. Strategies included well-planned recruitment, ongoing and appropriate supervision, reasonable adjustments/flexible work arrangements and self-care. Selective approaches tailored for the peer workforce as well as more universal ‘whole of workforce’ measures were raised.

#### 3.4.1. Recruitment

Adequate planning and recruitment was viewed as critical to organisational preparedness and key to ensuring an effective peer workforce. It was stressed by many that recruitment involved much more than selecting people purely on the basis of their mental health diagnosis. Participants described effective recruitment processes as assessing whether applicants had a range of ‘softer skills’ including advanced communication and the ability to navigate complicated situations. For some, a background or qualification in mental health was desirable. Other participants described an emphasis on considering the ‘whole person’ during recruitment:

We really want people to come in with all their quirks and bits and bobs that make them interesting, because we think the work we do is the work of relationships and people actually have much better relationships with humans than they do with ‘roles’

—Matt

Employment of peers was discussed by some participants as a ‘learning curve’, noting the time it had taken to effectively integrate a peer workforce. Better systems were deemed to evolve with time and ‘false starts’ were considered to contribute to necessary learning. Participants further described recruitment processes becoming more refined as the organisation understood peer roles better:

…as we’re recruiting new people we’re asking more questions, we’re a little bit smarter and it’s something that you learn over time... we now know what the right questions are to ask people.

—Bruce

Conversely, the consequence of recruiting people who may not be suitable for peer work was seen by some participants as a key impact on the perceived value of the role. Poor planning was seen to contribute to situations where peer workforce development appeared rushed and where the foundations for understanding and communication were not well established:

I think it was very much ‘we’re getting this money to do this, it’s gonna be great to have this fresh idea’ …rushed like a bull out a gate—just get people in positions and not actually think about whether they’re right for that position

—Octavia

#### 3.4.2. Flexible Workplaces/Reasonable Adjustments

For some participants, there were tensions around the idea of reasonable adjustments and a view that perhaps reasonable adjustments went too far. However, other participants believed the intention was not to over-protect the peer workforce, and that a good balance had been found at their organisation; supporting the workforce rather than ‘coddling’ peers. Peer executive/senior manager Alex, expressed an opinion that peer workers did not need adjustments different to other workers when the working environment was flexible and supportive:

…there is reasonable adjustment, we haven’t had to use that a great deal or call on it a great deal, cos I think people feel fairly supported and in a flexible working environment anyway…I don’t think any of us want to be wrapped in cotton wool and ‘kiddied’ through our roles, because that detracts from the roles

—Alex

Other participants regarded reasonable adjustments as a given as part of good staff management for any roles. Workforce structures and/or policies that were supportive of staff were widely considered by many participants to be of benefit for any employee:

All of our support structures need to support all of our staff … because who’s to say a person doesn’t have a lived experience or a person does have a lived experience?

—Josh

Other participants agreed with a whole of organisational approach and saw it as a means of combating prejudicial attitudes towards people with a mental health diagnosis:

…when you shift the frame and you’re introducing [support or policies] in terms of a whole of organisation, what you’re actually doing is de-stigmatising as well

—Penny

#### 3.4.3. Supervision

Most participants saw adequate and appropriate supervision as key to management of peers. Participants described a variety of supervision approaches including formal and informal supervision, with an emphasis on ensuring timely debriefing and support. ‘Open door policies’ were often raised in the context of informal supervision, as was the need for ongoing reflection and feedback. A sound understanding of recovery was deemed essential in the supervision of peer workers. Other qualities of good supervision were described as: empathy, experience with and understanding of peer workers, and significant grounding in organisational processes, government policies, and mental health in general. Some participants also considered supervision of peers by peers as necessary, particularly in relation to role clarity and mentoring on how to effectively use ‘lived experience’ effectively in work practice.

Supervision was not only seen as important to assist and support peers, but also to ensure the organisation was accountable to their peer staff, providing a work environment in which peers can be effective:

…it’s not only is the worker doing okay but it’s also is the organisation doing okay by the worker, and I think supervision is a medium whereby that at least can be checked

—Pippa

## 4. Discussion

### 4.1. Value of Role: Benefits of Peer Roles

Benefits to organisations and colleagues in traditional roles reflected themes in the wider literature, with participants viewing peers as essential to ensuring recovery orientation and enhancing person-directed care, as well as reducing service costs [6,7]. Also supporting earlier research findings, participants described peers’ empathy and ability to communicate effectively leading to improved understanding from co-workers in traditional roles [13]. Of interest, the benefits for service users described by participants focused almost exclusively on recovery and psychosocial outcomes, rather than clinical or medical outcomes. While the research findings align with other qualitative studies, this research has brought the ‘new’ voices and perspectives of management to thinking and evaluating peer roles and, in the light of this, arguably strengthens the evidence base for the value of the role in mental health services. Further research to explore these valuable perspectives would be beneficial, however funding to conduct such research—particularly led by peer or survivor researchers—has been and remains scarce [22].

Benefits for service users were reported as even more prevalent and this study provides additional support for earlier findings, including the peer propensity for more equitable relationships and assisting to address power imbalances within the service system [9]. Peers were viewed by participants as a living example of hope and recovery. As described within the peer discipline of ‘Intentional Peer Support’ the mutuality between peer workers and service users was likewise viewed as an impetus for shifting beyond limiting ideas of self that can develop as a result of service use [10].

Participants described their experiences managing and getting to know peer workers positively impacting their own views of service users, affirming previous research claiming exposure to people who are ‘out’ about their lived experience fosters greater understanding and empathy [15]. Having designated peer roles in the workplace was seen to increase understanding and alleviate the fear, assumptions, and myths surrounding people with mental health diagnoses.

While this familiarity with peer workers who are out about their mental health experiences may be impacting positively on prejudicial attitudes towards service users, the lived experience of people in traditional mental health roles is still largely under-utilised and often considered ‘unprofessional’ to disclose [23]. Some organisations are embracing the lived experience of staff in traditional roles by including lived experience as a desirable quality within position descriptions and encouraging people in traditional roles to apply for newly created peer positions [24], however these practices are still not usual. The fear to disclose or openly utilise lived experience is still prevalent for many employed in traditional roles [25] and provides a barrier to best practice therapeutic relationship building and recovery outcomes for service users [24]. Participants shared their opinion that workers in traditional roles were at increased risk of burnout and absenteeism when unwilling or unable to disclose and receive support for their mental health challenges. It could be argued that while discriminatory attitudes still exist towards people with a lived experience in traditional roles, services cannot claim to be truly free from prejudice towards service users. Given the peer impact on prejudicial attitudes, and understanding of working from a lived experience perspective, peer staff may be a valuable asset in both encouraging organisations to embrace the lived experience of staff in traditional roles, and assisting people in traditional roles to use their lived experience in meaningful and effective ways.

### 4.2. Limitations of Peer Roles and Strategies to Maximise Benefits

Participants discussed the preconception of frequent absenteeism/burnout and the challenges when an organisation or individual previously had a negative experience employing a peer worker. Differences were noted, with participants who had greater experience managing a peer workforce more likely to attribute these challenges to organisation or planning error, rather than as a limitation in the peer role itself. The literature highlights the need for meaningfully thought out positions and role clarity to maximise the unique benefits of peer roles [26]. Lack of organisational consideration of the purpose of peer positions has been found to contribute to poor role clarity [27] and studies have emphasised the need to not attempt a ‘one size fits all’ position descriptor, rather reflecting the diverse approaches available [26]. Just as existing literature has acknowledged the importance of supervision in maintaining role clarity, [28,29] participants repeatedly acknowledged the role of both traditional and peer supervision in effectively sustaining peer workers; guarding against burn out; and as a platform to reflect on the nature of peer work, including potential challenges.

Generally, there is evidence that workplace accommodations to meet the needs of individual workers may be beneficial, particularly when there are limited natural supports present in the workplace and, in these cases, they have been linked to longer tenure [30]. Workplace adjustments are less focused on the physical environment of an organisation and more on human resource management [31]. However, overall, little is known about what reasonable accommodations are suited to employees with mental health challenges [32]. Significant variety was found among participants in regard to reasonable adjustments as a desirable strategy for supporting peer workers. While adjustments can address specific needs, and were favoured by a few managers in this study, the need for a whole service approach to staff support, equity, and flexibility was more frequently raised by participants. Legislation and workplace policies are effective in building an integrative approach that mitigates risks of discrimination and marginalisation for peer workers [33], however diversity management research reports formal structures are only partly effective [34], with the involvement of managers and leaders being seen as critical [35]. The literature on workplace diversity and inclusion also points to the diversity education of all staff as a strategy which supports longer and more successful employment of people in diversity roles [36], consequently suggesting the need for integrative multi-level organisational approaches to facilitate authentic integration of peer workers [37].

Parallels in respective literature and a lack of an existing body of knowledge on how to manage and support peers suggests mental health peer work may benefit from implementation and support in the context of broader organisational diversity management and inclusion strategies. Positioning peer roles within diversity management may also promote mainstream credibility for the roles and encourage the potential role of peers outside mental health service systems, due to the longer term, wider scale acceptance of diversity employment amongst management in the wider workforce. While diversity management is not without its limitations, exploring the potential for such an approach may be further warranted by evidence that successful diversity management is linked to workplace performance [38], job satisfaction, and higher wellbeing for staff [39].

### 4.3. Limitations of the Study

This study, as with most qualitative research, is limited by relatively small numbers, and to only one state in an English-speaking country. Furthermore, racial and ethnic diversity was not included in the demographic questionnaire and is therefore not represented in the results. Transferability and cultural representation is therefore limited. Further exploration of the appropriateness of diversity management concepts and policies to peer work may also be of assistance in drawing attention to the need for culturally inclusive and diverse research in the area of peer support.

## 5. Conclusions

Participants identified many benefits of peer work encompassing several key stakeholders—organisations, colleagues in traditional roles, and service users. Benefits for organisations included enhanced recovery orientation and a whole of person focus. Participants also reported improved therapeutic relationship building for colleagues in traditional roles as a result of deepened empathy and understanding of service users. Service users were reported to have experienced reduced hospitalisation and service use, and benefitted from increased hope, empathy, mutuality, and more equal power dynamics. The reported limitations of peer work were often mitigated by strategies that ensured meaningful, merit-based recruitment and ongoing support for roles in the form of role clarity, appropriate supervision, and flexibility in the workplace. Parallels were found between the issues raised by participants and diversity management literature. While peer work is not commonly considered ‘diversity employment’, the question is posed whether it may be useful to draw from and/or position peer work within the diversity and inclusion agenda to aid effective understanding and management of peers.

## Figures and Tables

**Table 1:** Participant Roles and Numbers.

No. of Participants	Type of Role Participants Were Employed in
11	Traditional executive or senior management roles within not for profit organisations
8	Traditional executive or senior management roles within government organisations
5	Designated peer executive or senior management roles within not for profit organisations
3	Designated peer executive or senior management roles within government organisations
2	Designated carer executive or senior management roles within government organisations
